# A systematic review of cognitive remediation therapy for anorexia nervosa – development, current state and implications for future research and clinical practice

**DOI:** 10.1186/s40337-014-0026-y

**Published:** 2014-09-10

**Authors:** Camilla Lindvall Dahlgren, Øyvind Rø

**Affiliations:** Regional Department for Eating Disorders, Division of Mental Health and Addiction, Oslo University Hospital, Ullevål HF, Oslo, Norway; Department of Psychiatry, Centre for Research on Eating Disorders at Oxford (CREDO), University of Oxford, Warneford Hospital, Oxford, UK; Division of Mental Health and Addiction, Institute of Clinical Medicine, University of Oslo, Oslo, Norway

**Keywords:** Cognitive remediation therapy, Anorexia nervosa, Review, Treatment, Neuropsychology, Eating disorders, Metacognition

## Abstract

**Objective:**

To systematically review studies of cognitive remediation therapy (CRT) for anorexia nervosa (AN), and to discuss findings with references to clinical practice and future research.

**Method:**

The literature was reviewed using the PubMed, Web of Science and PsycINFO search terms “cognitive remediation therapy” AND “anorexia nervosa”. Papers published online between 2005 and 2013 were selected on the basis of three inclusion criteria: 1) studies of any design focusing on CRT for AN, 2) papers that were written in English or had an available published English translation, and 3) papers published in peer reviewed journals.

**Results:**

A total of 45 papers were identified of which 21 were recognized as being relevant for the review. Relevant papers were divided into three different categories 1) single case reports, 2) case series and 3) randomised controlled trials (RCTs). Single case studies and case series yielded strong evidence of feasibility and acceptability of CRT for AN despite great variety in sample compositions. Four RCTs demonstrate that CRT has the potential of enhancing the effectiveness of current treatments, reduce attrition, increase cognitive set-shifting abilities and quality of life, as well as reduce eating disorder psychopathology.

**Discussion:**

The number of CRT studies published is growing rapidly, in particular RCTs. Further research is needed to define the primary aim of delivering CRT to patients with eating disorders, and to establish how to best measure the effect of the intervention. Moreover, researchers and clinicians should focus on identifying and assessing specific versus non-specific CRT contributions, and explore long-term effects of the intervention. It is imperative that adolescent RCTs are conducted to evaluate how CRT may be effective as a treatment for this young patient group.

## Introduction

Anorexia nervosa (AN) is a multifaceted mental disorder characterized by pathological eating behaviours. It is a serious condition and in some cases, even life threatening [1] with significant psychiatric and physical co-morbidity, and poor quality of life [2,3]. Symptoms of the illness most commonly occur in early to late adolescence, and has a prevalence rate of 0.5-0.7% among adolescent females between 15 and 19 [4]. In the DSM-5 [5], AN is characterized by distorted body image and excessive dieting that leads to severe weight loss, with a pathological fear of becoming fat.

Although previous research in the field of eating disorders (EDs) has made important contributions to the understanding of AN, the evidence base for treatment efficacy in adult AN is scarce [6]. For younger patients, current research evidence suggests that a particular form of family based therapy, FBT [7], is the most efficient approach [8–10]. Despite numerous approaches currently being applied in the treatment of the disorder such as cognitive behavioural therapy (CBT), interpersonal therapy (IPT), cognitive analytic therapy (CAT), family based therapy (FBT) (for children and adolescents), nutritional counselling etc., existing randomised controlled trials (RCTs) are limited [11]. Also, those existing appear inconclusive as short vs. long-term investigations, sub group phenomena and current state of illness appear to influence treatment outcome, making it difficult to compare studies and predict long-term effects.

In recent years, there has been a substantial increase in papers investigating the impact of neuropsychology on eating disorder (ED) aetiology-, maintenance-, and recovery. Clinical neuropsychology is concerned with the applied science of brain-behaviour relationships, and in AN, the focus has been primarily to establish the extent to which weaknesses in cognitive flexibility (i.e. the inability to shift or change mental and behavioural strategies) and central coherence (i.e. the preoccupation with details at the cost of global/contextual processing) contribute to the development of the illness, its perseverance and the likelihood of recovery [12–16]. Early neuropsychological studies and clinical observations of adults with AN laid the groundwork for the development of cognitive remediation therapy (CRT), an intervention specifically tailored to remedy weaknesses in these two domains (i.e. cognitive flexibility and central coherence), and designed to encourage patients to reflect on their thinking styles. The CRT technique was originally conceptualized and developed from patients suffering from brain lesions [17], but over the last 50 years, the method and its application has been gradually adapted to suit individuals suffering from other health conditions as well. In psychiatry, CRT has been most commonly associated with the investigation of cognitive dysfunction in patients with schizophrenia, and dating back to the early 1990’s, there is an extensive body of work describing positive outcomes of CRT for this patient group [18,19]. The approach has also been successful in treating other mental health conditions such as mood disorders [20] attention deficit hyperactivity disorder (ADHD) [21], alcohol dependence [22], geriatric depression [23] and obsessive compulsive disorder (OCD) [24].

CRT for AN is a relatively new treatment approach. For this patient group, the intervention has been delivered as an addition to treatment as usual, and focuses on the process of thinking (i.e. the how) rather than the content (i.e. the what). In contrast to traditional interventions that centre on increasing food intake and on addressing ED specific symptoms such as weight and shape concerns, CRT aims neither to address nor directly treat these. The focus is primarily to decrease rigidity (i.e. increase flexibility) and achieve a balance between local (detailed) and global (the bigger picture) information processing strategies.

The very first paper on CRT for AN was published in 2005 [25], and reports results from a single case study illustrating the use of CRT for an adult female inpatient refusing to participate in the core treatment approaches offered. The materials used were hand-picked from the set-shifting module in Delahunty & Morice’s “*A training programme for the remediation of cognitive deficits in schizophrenia*” [26], and gradually thereafter, novel materials were developed and assembled in CRT manuals specifically tailored to remediate cognitive weaknesses in females with AN (e.g. [27]). Research on CRT for AN is evolving rapidly, and is currently transforming from feasibility and piloting trials into the realm of what is considered to be the gold standard when it comes to testing the effect of a specific treatment approach; RCTs. Alongside study design transformations, the aim of delivering CRT and contributions thought to be associated with the intervention also appear to have changed. CRT was initially hypothesized to improve thinking skills beneficial for every day living, and to prepare patients for therapeutic interventions focusing on thought, belief and emotion. However, during the course of its development and adaptation, exploration of potential intervention outcomes have expanded considerably, and now also include ED psychopathology, comorbidity, perfectionism, treatment attrition and comparisons to other neurocognitive interventions. As knowledge of the applicability of CRT for AN has accumulated during almost a decade, research and clinical work has now guided a small number of RCTs, and for the first time, researchers have been able to demonstrate the effect of the treatment.

Three papers have sought to describe the current evidence of CRT for AN, and its future directions [28–30]. The first of these was published seven years ago [28] (merely two years after the first CRT for AN paper) and lacks a current update of the field. The second paper [29] describes how CRT for AN has been developed and refined, and suggests future research directions, but lacks a systematic overview of studies published in this field. The most recent summary paper published in 2013 [30] also aims to provide the reader with current evidence and future research directions, in addition to presenting an overview of published studies reporting CRT for AN. However, merely nine studies are included in this overview, leaving out a total of 12 studies published in this field, four of which are recently published effect studies (i.e. RCTs). As the intervention itself, as well as the assessment of its efficacy has gone through major recent developments, the current paper aims to contribute to the field further by presenting a systematic overview of *all* studies of CRT for AN. Doing so, the authors wish to add to the field by delineating the interventions developmental trajectory in both adults and adolescents, its current state, and it’s potency in future treatment of AN.

## Review

The literature was reviewed using the PubMed, Web of Knowledge and PsycINFO search terms “cognitive remediation therapy” AND “anorexia nervosa”. The PubMed search yielded 27 publications, Web of Science 23 publications and PsycINFO 25 publications, all published online between August 2005 and November 2013. Together, the three searches yielded 75 publications, which were cross-referenced. After duplicates had been omitted, 45 unique publications were identified. Publication titles and abstracts were screened initially, and eligibility was established by reading the full texts. A manual reference search was conducted (i.e. references lists in papers from the initial database search were examined) to explore additional papers of relevance. One additional paper was identified during this search [29]. On the basis of the inclusion criteria outlined below, 21 papers were identified as being relevant, and selected for review.

### Inclusion criteria

1. Studies of any design focusing on cognitive remediation therapy and AN.
2. Articles that are written in English or has an available published English translation.
3. Articles that are published in peer reviewed journals.

## Results

### Excluded studies

**Figure 1:**
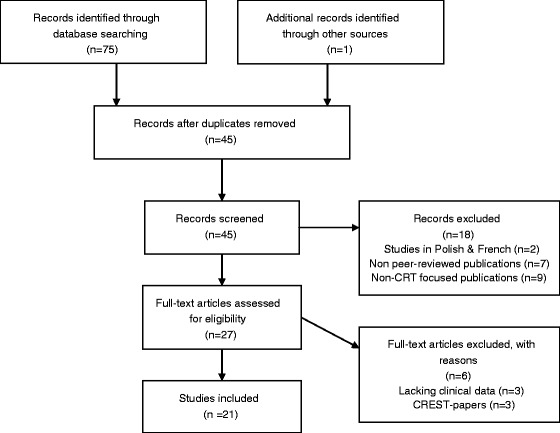
**Search strategy used to identify and screen studies for review.**

All in all, 24 publications were excluded from the review. Two CRT studies were excluded as they were written in Polish [31] and French [32], and one additional CRT publication was omitted as it had been published as a meeting abstract rather than in an article format [33]. Nine studies were excluded as these did not focus specifically on CRT for AN [34–42], and six publications were excluded as these represented either books or book chapters [43–48] rather than peer reviewed papers. Three studies evaluating the combination of cognitive remediation therapy and emotional skills training (CREST) [49–51] were excluded from the review as the design of these studies did not allow for assessment of CRT specific contributions. Finally, three theoretical CRT papers lacking clinical data were omitted [28–30]. The search strategy used to identify and screen studies for review is presented in the PRISMA flow diagram (Figure 1).

### Overview of included studies

The 21 reviewed studies were published online between August 2005 and November 2013, with sample sizes ranging from 1 to 46 participants, and ages ranging from 12–62. Eleven studies explore CRT in adult samples (ages 18–45), six studies explore CRT in adolescents (ages 12–19), and the remaining four studies samples mix adolescent and adult patients (ages 14–62). With the exception of seven male participants, all patients included in the studies (receiving CRT) were female (N = 355), and the vast majority of participants were diagnosed with AN. One study had included patients with a current diagnosis of AN plus recently recovered AN patients (N = non specified) [52], and another study had included both current AN (N = 17) patients as well as participants with an eating disorder not otherwise specified (EDNOS) diagnosis (N = 7) [53]. Three of the 21 publications were based on the same sample [54–56]. The authors are not aware of additional sample overlaps. One of the most recent studies explores transdiagnostic CRT without specifying ED diagnosis [57]. Out of the 21 studies reviewed, four reported results from RCTs, all which were published online in 2013 [57–60]. Participants in the included studies were currently enrolled in either in- or outpatient treatment for their ED, and all received CRT as a conjunctive intervention. Included studies varied widely, for example according to whether CRT was delivered individually, in groups or families, the number of sessions offered, the intensity of these and the materials used. Studies also varied according to the aim of the study and consequently, what outcome measures were applied. Three different types of CRT publications were identified:

1. Single case studies (n = 3).
2. Case series (n = 14).
3. RCTs (n = 4).

The main characteristics of the publications are presented in Table 1. Publications are stratified according to *publication type* and *chronological order*.

**Table 1:** **Characteristics of included studies**

**SINGLE CASE STUDIES**									
**Year author**	**Aim of study**	**N**	**Diagnosis**	**Age**	**Setting**	**Mode of delivery**	**No. of sessions**	**Intensity**	**Results**
2005 Davies & Tchanturia	To illustrate how CRT can be used to stimulate mental activities and improve thinking skills.	1	AN	21	Inpatients	Individual	10	Two 3 weekly sessions + 2 twice weekly	Improvement in cognitive set-shifting.
2006 Tchanturia et al.	To demonstrate the potential benefits of CRT through a case-report.	1	AN	42	Inpatients	Individual	10	Once or twice weekly	The authors propose cognitive flexibility training as a pre-treatment intervention for treatment resistant inpatient cases.
2007 Pretorius & Tchanturia	To demonstrate how CRT has been adapted for AN through a case-report.	1	AN	31	Inpatients	Individual	10	Twice weekly	Increase in BMI, tasks were a bit repetitive but the patient was able to develop new flexible strategies for implementation in real-life settings.
**CASE SERIES**									
**Year author**	**Aim of study**	**N**	**Diagnosis**	**Age range (Mean)**	**Setting**	**Mode of delivery**	**No. of sessions**	**Intensity**	**Results**
2007 Tchanturia et al.	Explore the effect of CRT in set-shifting and investigates in acceptability for AN patients.	4	AN	21-42	Inpatients	Individual	10	Not reported	Improved cognitive flexibility and positive feedback for CRT.
2008 Whitney et al.	To examine patients’ experience of participating in CRT.	21	AN	17-54 (30.3)	Inpatients	Individual	10	Once or twice weekly	Positive that the intervention did not focus on food, helpful in reducing perfectionism and rigidity. More difficulty in tasks wanted, and help to implement newly learned skills.
2008 Tchanturia et al.	To explore neuropsychological task performance before and after CRT.	27	AN	(28.8)	Inpatients	Individual	10	Twice weekly	Improvements in cognitive performance on the Brixton & CatBat tasks. No sign improvements in other neuropsychological tasks. Significant decrease in depression.
2010 Genders & Tchanturia	To report CRT development and acceptability in a group format (2 male participants).	30	AN	14-62	Inpatients + Outpatients	Group	4	Once weekly	Statistically significant gains in self-reports of ability to change. CRT was found to be acceptable, useful and positive by both patient and group facilitators.
2010 Pitt et al.	To evaluate the benefit of CRT in terms of changes in self-reported perfectionism and patient feedback.	7	AN + Recovered AN	(29.5)	Outpatients	Individual	10	Once or twice weekly	Initially confusing but mentally stimulating. Increased awareness of ones own thinking style. Both higher and lower degrees of perfectionism post CRT.
2011 Wood et al.	To describe group CRT for adolescents.	9	AN	13-19	Inpatients	Group	10	Once and twice weekly	Patients found CRT fun and playful, helped develop a positive therapeutic relationship. Negative feedback involved tasks being boring, too easy tasks, repetitive, some negative group dynamics affected the group work.
2011 Easter & Tchanturia	To examine how CRT has been implemented in the daily life of the patients through therapist feedback letters.	26	AN	Adults	Inpatients	Not reported	10	Twice weekly	Feedback letters were positive and motivational, and highlighted challenges in metacognitive ability and in transferring in therapy skills to every day life for the patients.
2012 Abbate-Daga et al.	To measure the effect of CRT on cognitive flexibility.	20	AN	(22.5)	Outpatients	Individual	10	Once weekly	Improved neuropsychological performance. Significant improvement on impulse regulation, interoceptive awareness reflexive skills and awareness.
2012 Pretorius et al.	Evaluation of group CRT for adolescents with AN through self-reported flexibility and motivation.	30	AN + EDNOS	12-17 (15.6)	Outpatients	Group	4	Once weekly	No significant changes in flexibility and motivation to get better. Patient feedback: “interesting”, “fun”, “not too demanding”, “helpful”, but also “dull” and “repetitive”. Patients wanted more variation in exercises.
2013 Zuchova et al.	To explore the feasibility and acceptability of group CRT for inpatients with AN.	34	AN	18-45	Inpatients	Group	10	Once a week	Group-based CRT could be well incorporated into the therapeutic program at the eating disorders unit, and was well received by the participants.
2013 Dahlgren et al. (a)	To assess the feasibility of CRT for children and adolescents with AN.	20	AN	13-18 (15.9)	Inpatients + Outpatients	Individual	7-12	Once or twice weekly	Results indicate feasibility for young patients with AN with regards to recruitment, materials, individual tailoring and delivery, and clinician feasibility.
2013 Dahlgren et al. (b)	To assess neuropsychological functioning pre and post CRT.	20	AN	13-18 (15.9)	Inpatients + Outpatients	Individual	7-12	Once or twice weekly	Significant changes in weight, depression, visio-spatial memory, global information processing and verbal fluency. Changes in weight had a significant effect on improvements in visio-spatial memory and verbal fluency.
2013 Dahlgren et al. (c)	To explore self-reports and parental ratings of executive function before and after CRT.	17	AN	13-18 (15.9)	Inpatients + Outpatients	Individual	7-12	Once or twice weekly	Decrease in patient BRIEF shift subscale post CRT. Parent reports revealed significant lower scores on shift-, emotional control- and working memory subscales, and on two composite indices.
2013 Lask & Roberts	To assess feasibility of CRT in family settings.	4	AN	14-19	Inpatients + Outpatients	Family	01/06/14	Varying from weekly to monthly	CRT is useful when applied in families, and authors suggest a subsequent formal evaluation of this mode of delivery.
**RANDOMISED CONTROLLED TRIALS**									
**Year author**	**Aim of study**	**N**	**Diagnosis**	**Age range (Mean)**	**Setting**	**Mode of delivery**	**No. of sessions**	**Intensity**	**Results**
2013 Lock et al.	To evaluate the feasibility of using CRT to reduce attrition in RCT’s for AN.	23*/23	AN	(22.7s)	Outpatients	Individual	8	8 sessions during 2 months	CRT is acceptable and feasible for use in RCTs. It may also reduce short-term attrition.
2013 Brockmeyer et al.	To investigate feasibility and efficacy of specifically tailored CRT, compared to NNT.	11*/14	AN	(23.6*/26.7)	Inpatients + Outpatients	Computer assisted & Individual	30	30 sessions over 3 weeks	Participants receiving CRT outperformed participants in the NNT condition in cognitive set-shifting. Both groups showed high treatment acceptance.
2013 Steinglass et al.	To evaluate AN-EXRP as an adjunctive strategy to improve eating behaviour during weight restoration.	15*/15	AN	16-45	Inpatients	Individual & Group	12	3 times a week over 4 weeks	AN-EXRP is associated with better caloric intake than CRT when assessed through laboratory meals.
2013 Dingemans et al.	To investigate the effectiveness of CRT in a randomised controlled trial comparing treatment as usual (TAU) and TAU plus CRT.	41*/41	ED	17-53	Mainly inpatients	Individual	10	10 sessions over 6 weeks	CRT plus TAU was superior in terms of ED-related quality of life and ED psychopathology. CRT appears to be promising in enhancing effectiveness of concurrent treatment.

*Note.* Results are presented descriptively due large discrepancies between studies, and for some papers, due to the lack of quantitative data.

* = Interventions details (i.e. mode of delivery, session details, intensity and intervention materials) refer to the CRT arm only.

*AN* = Anorexia Nervosa; *AN-EXRP* = Exposure and Response prevention for AN; *BMI* = Body Max Index; *CRT* = Cognitive Remediation Therapy; *ED* = Eating Disorder; *EDNOS* = Eating Disorder Not Otherwise Specified; *NNT* = Non-specific Neurocognitive Training; *RCT* = Randomised Controlled Trial.

#### 1) Single case studies

The three single case studies [25,61,62] represent the very beginning of the development of CRT for AN. All papers describe a single, adult female inpatient with longstanding AN, undergoing 10 sessions of individual CRT. The three papers illustrate how the CRT materials were adopted and adapted from the set-shifting module from Delahunty and Morice’s work on patients with schizophrenia [26], and how this material became the corner stone in the development of the CRT materials that most clinicians and researchers use today. The earliest of the three papers [25] brings about the importance of bridging the gap between in-session task performance and its relevance to everyday undertakings, a crucial part of CRT, which has been adhered to in the majority of subsequent studies. In the most recent of these three single case studies [62], the concept of behavioural tasks (in subsequent studies often referred to as *homework*) is introduced as a part of the therapeutic work with the patient, aiming to facilitate the implementation of newly learned strategies to everyday life situations. This study is descriptive in it’s nature, and focuses on patients’ experiences with CRT. It delineates the potential of CRT in increasing the effectiveness of other interventions, and with regards to the development of a heightened awareness of the patients’ thinking style. The two former papers [25,62] set out to investigate changes in neuropsychological functioning and clinical variables during the course of the intervention, and were identical in terms of outcome measures used. Results were inconclusive in that some performance measures had improved in one study, whereas they had not on the other study, and contrary, some measures did not improve in the first study, but did so in the latter.

#### 2) Case series

Three different types of case series were identified: feasibility studies, studies focusing on pre and post CRT assessments, and studies that overlapped with regard to these two categories.

#### 1. Feasibility studies

Six feasibility studies [55,63–67] were identified amongst the 21 papers. All studies investigated CRT for patients with AN, but the diversity of sample compositions e.g. sample size, age, in- and/or outpatients), intervention approaches (e.g. individual-, family- or group therapy, treatment intensity, intervention materials) and outcome measures (qualitative or quantitative, types of assessment instruments) rendered it difficult to compare studies and to generalize findings. Three papers reported results from patient feedback letters [65–67] with the two latter supporting the feasibility of CRT delivered in a group format, and the former when delivered individually. These three studies investigated feasibility for patients ranging widely in ages (13–54), strengthening the applicability of CRT across ages, and support the feasibility of the intervention at various stages of illness and developmental trajectories. The Wood et al. study [66] is the first to explore CRT for adolescents with AN, and it is also the first paper to present a detailed outline of the specific task(s) used each session. Results yielded evidence of feasibility and patient satisfaction, but also feedback on some of the drawbacks of delivering CRT in a group format (e.g. lack of relevance and unfavourable group dynamics). In 2013, the second paper exploring CRT for adolescents with AN was published. This time, the format of delivery had changed, and the 20 patients in the Dahlgren et al. study [55] received individually delivered CRT, tailored to meet each patient’s specific needs. This was the first study to use materials developed specifically for children and adolescents [68]. The study published by Easter and Tchanturia [63] illustrates a novel approach to feasibility in its application of *therapists’* feedback letters to examine how CRT has been implemented in the daily life of the patients. It is pioneering and unique in its attempt to establish a more ecologically valid interpretation of how CRT affects the daily life of the patients. No subsequent studies have used this type of methodology, although many report using feedback letters. The most recent feasibility study explores the use of CRT when used in family settings [64] and reports clinical and observational data to illustrate its use in this novel mode of delivery. Results supported the acceptability for both patients and their caregivers, and the intervention appeared to increase motivation to change in the patients.

#### 2. Studies assessing pre and post CRT functioning

Four studies investigate pre and post CRT functioning without using control groups [54,69–71], and illustrate how the use of a wide variety of outcome measures have been applied to investigate changes across the intervention span. Such investigations include ED psychopathology, comorbidity, impulse regulation, executive functions (including cognitive flexibility and decision-making), central coherence, and overall functioning. Similar in the four studies is that the patients appear to have received approximately the same number of sessions (i.e. 10). However, comparing the studies it is clear that contrasting features outnumber similarities in terms of age, treatment intensity, the CRT materials used and the choice of assessment instruments. Results also reveal how different aspects of the same measure have been used to evaluate changes post CRT. For example, both Tchanturia et al. [72] and Abbate-Daga et al. [59] found significant changes in Trail Making Test (TMT) [72] performance in adult patients with AN, whereas Dahlgren et al. [54] did not in adolescents with AN. However, results from a variety of TMT measures such as shifting time-, total completion time- and attention were reported in the different papers, rendering it difficult to compare results. Three studies [54,56,71] (of which the two latter are based on the same sample) report a decrease in depression post CRT, with one adolescent study [54] challenging the previously held notion that weight restoration alone does not significantly contribute to changes in neuropsychological functioning [25,71]. Further, the two studies published by Dahlgren et al. [54,70] address methodological issues previously ignored, such as test-retest effects and biases associated with the absence of proper controlling and corrections for multiple comparisons. In Dahlgren et al. [70], a novel approach to investigate pre and post CRT functioning is being used: the self-report measure “Behavior Rating Inventory of Executive Function” (BRIEF) [73]. In this paper, the authors compare patient and parental reports of executive functioning (EF) before and after CRT. On a group level, conclusions point in the direction of scorings within the normal range, and yields results of substantial discrepancies between parent- and patient’s ratings of executive functioning. On an individual level however, large within-group differences emerge.

#### 3. Overlapping studies

We identified four studies with a distinct overlap with regards to feasibility and pre and post CRT assessments without control groups [52,53,74,75]. The least recent publication from 2007 [74], although using a very small sample (N = 4), represents the transition from the single case study format, to a novel study design where larger groups of patients are included, and where authors set out to investigate both feasibility and neuropsychological performance before and after the CRT. Observed significant changes in set-shifting and patient feedback from this study was used to further develop CRT for patients with AN. Also, results from this study led to the refining of materials, and the inclusion of behavioural task in the updated [in-house] manual produced a few years later [Tchanturia & Davies: Cognitive Remediation Programme for Anorexia Nervosa: A Manual for Practitioners, Unpublished]. Group CRT was first described and evaluated in the study by Genders & Tchanturia [74] and similar to the Tchanturia et al.’s work from 2007, this study also sought to combine knowledge of feasibility and changes post CRT. Results supported acceptability for both patients and group facilitators, and heightened rates of self-reported ability to change were reported by the patients. No significant changes in cognitive flexibility or self-esteem were reported. When the second paper on group-CRT was published in 2012 [53] tasks had been adapted from the Genders & Tchanturia study mentioned above, but to accommodate the patients who were already engaged in an intense six-week programme, no more than four sessions were offered. Again, similar to the previous group CRT study, results supported the acceptability of the intervention, but did not support significant changes in self-reported flexibility. In 2010, Pitt et al. [52] made an important addition to the field in not only by exploring the feasibility through patient feedback, but by introducing a novel CRT outcome measure; perfectionism. As the first of its kind, this study provided data based solely on outpatients assessments. Similar to the data reported in Tchanturia et al. in 2007 [75], self-reported flexibility was significantly higher post CRT. In terms of perfectionism, results could not be calculated statistically due to the small sample (N = 7), and trends were inconclusive as both increases and decreases in scores were observed.

#### 3) RCTs

The four most recent studies on CRT for AN are all RCTs in adults, and represent a giant leap from that of feasibility trials and case studies in terms of its potency of measuring intervention effects. The first of these studies [59] explores the role of CRT in reducing attrition in RCTs for AN. In this study, 46 patients with AN were randomised to receive either eight sessions of CRT or CBT over a period of two months. Following these initial eight sessions, both groups received 16 CBT sessions over a period of four months. For patients receiving CRT, results revealed lower dropout rates compared to the CBT group (13 and 33% respectively), as well as significant changes in set-shifting and central coherence at the end of the intervention. However, dropout rates were obtained through data collected at the main outcome point (session eight), after which the rate of attrition increased in the CRT group and eventually, rose to a level matching that of the CBT patient group. Also, no significant differences between CRT and CBT groups in outcome measures such as weight or ED psychopathology were observed at the end of treatment. In addition, results indicate that CRT has an effect on specific neurocognitive functions, but shows no superiority in terms of improving ED symptomatology compared to CBT. Somewhat later in 2013, Brockmeyer et al. [58] introduced a novel approach to CRT; face-to-face sessions *plus* computer assisted homework. Data from 25 treatment completers, 11 receiving CRT focusing specifically on cognitive flexibility, and 14 receiving non-specific neurocognitive therapy (NNT), was analysed. Similar to the study conducted by Lock and colleagues [59], no significant difference in treatment adherence was observed. However, in line with Lock et al.’s initial RCT [59], Brockmeyer et al. [58] also found changes in cognitive flexibility being significantly higher in the group receiving CRT, than in the control group (i.e. NNT). In November 2013, two additional RCTs were published (online) [57,60]. The first, a small study conducted by Steinglass and colleagues [60], sought to evaluate the effect of Exposure and Response Prevention for AN (AN-EXRP) on eating behaviour during weight restoration, by comparing it to CRT. Data from 15 inpatient treatment completers was analysed, and yielded evidence of superior caloric intake in the AN-EXRP arm compared to CRT, with improvements that were significantly associated with reduced eating related anxiety. The most recent RCT [57] is also the largest with 82 patients receiving either treatment as usual (TAU) (N = 41) or CRT plus TAU (N = 41). It is also the first transdiagnostic CRT study, exploring the effect of the intervention in a sample of ED patients without reporting specific ED subtypes. Results revealed that patients in the CRT plus TAU arm reported higher levels of ED-related quality of life at the end of treatment than those who received TAU alone, and also, patients with poor baseline set-shifting abilities benefited more from CRT than those performing within the norms a this time point. Dropout rates in the two groups were nearly identical at 6-month follow up (approximately 20%).

## Discussion

To our knowledge, this is the first paper reviewing the whole literature for CRT for AN, and the first paper delineating its developmental trajectory from single case studies to RCTs. Because of the preliminary nature of case reports and case series, and the great variety amongst them, it is difficult to compare and generalize results. Methodological challenges such as short test-retest spans and its associated risk of learning effects, the use of identical pre and post CRT assessment instruments and the lack of control group renders it impossible to conclude whether observed changes (or the lack of these) are direct results of the intervention itself, or associated with other factors such as changes in comorbidity, ED psychopathology, or weight. However, results from these studies suggest that individual CRT appears to be associated with lower dropout rates than group CRT, and has the potential of creating a positive patient- therapist alliance [76]. Further, group therapy appears to appeal to patients in increasing awareness of shared cognitive styles, but has also been reported fostering negative group dynamics, and tasks assigned during the therapy is not always perceived as relevant [66]. At present, there is only one report of family CRT [64], and although it appears as family CRT strengthens the understanding of how cognitive styles affect family dynamics, improves communication and cooperation during treatment, one must stress that family CRT should not replace existing treatments, but rather be seen as a conjunct to ordinary treatment engagements. Overall, single case studies and case series presented in this paper support the feasibility of CRT across ages, illness severity, current treatment engagements and when delivered in a number of different formats.

As evident from Table 1, the mode of CRT delivery is changing rapidly, and as new modalities are described, tested and evaluated, it is crucial to attempt comparing results so as to understand the advantages and disadvantages of each approach. With the emergence of RCTs, the potential of measuring the effectiveness of CRT has increased dramatically, and so has the possibility of comparing results.

The four RCTs presented in this paper, although varying in terms of study design and aims, depicts how CRT is currently being implemented in a variety of settings, with patients suffering from various forms of EDs, and through different means of delivery. The first two studies [58,59] support its efficiency in improving specific neurocognitive functions, whereas the first [59] and last [57] RCT published yielded evidence of its potency in reducing attrition and enhancing the effectiveness of concurrent treatment. In terms of moderator analyses, Lock et al. [59] failed to find evidence of weight and comorbidity as moderators for treatment response, whereas Dingemans et al. [57] showed that patients with poor baseline set-shifting abilities benefited more from CRT than those scoring within the norms. The emergence of the novel mode of delivery described in Brockmeyer et al. [58] exemplifies how face-to-face treatment in combination with computer assisted sessions might intensify learning and awareness, and how conjunctive CRT might help empowering patients to practice new skills using novel technologies.

The potency of CRT with regards to improvements in ED psychopathology and comorbid psychiatric illnesses still remains uncertain, and larger RCTs are needed to further address this issue. Also, as the mode of delivery and study aims in the four RCTs differ substantially, interpretation of results should be viewed as specific to each study rather than general, and comparisons should take into account the observed variation in study designs, mode of treatment delivery, choice of outcome measures and patient characteristics.

To date, adolescent CRT case studies- and series have supported the feasibility and acceptability of the intervention, both when delivered individually [53,55] in groups [67] and in families [65]. However, as all current RCTs are oriented towards adult patients, we still know little about the intervention’s potential in terms of efficacy in younger AN populations. Further, as many recent studies fail to detect neuropsychological impairment in adolescent AN patients [12,40,77–81], it might be timely to consider other components than neuropsychological performance when assessing the effect of CRT in this patient group, and to learn from recent results suggesting that CRT is more beneficent for those struggling with cognitive weaknesses than in those who perform within the norms [57]. For young AN patients, one might suggest revisiting one of the original aims of the intervention; preparation for subsequent treatment [25,61], but also to pay attention to results recently surfaced such as attrition reduction [59] and enhancing the effectiveness of concurrent treatments [57]. Further, as CRT aims to address thoughts and behaviours as they manifest in the patient’s everyday life, novel measures such as metacognitive abilities, quality of life or every day coping strategies might also be useful in assessing outcomes associated with the intervention. Finally, as evident from the papers presented in this paper, there are considerable discrepancies with regard to the aim of delivering CRT to patients with EDs. Although it is clear that CRT is still in its developmental phase and has followed a logic course of methodology (from single case studies to RCTs), the field would benefit greatly from finding a common ground on which the aim of delivering the intervention is based, and subsequently, using similar outcome measures to assess global intervention effects.

## Conclusion

The main conclusion of this review is that there is strong evidence of feasibility of CRT for AN across ages and illness severity. With the emergence of several recent RCTs, new evidence also suggest that that the intervention is effective in reducing attrition, enhancing the efficacy of concurrent treatment and improve cognitive set-shifting. To further strengthen our understanding of mechanisms of action in CRT, future studies should focus on (1) exploring the long-term effects of the treatment, (2) investigate the influence of moderators such as comorbidity (e.g. depression, anxiety and OCD), illness severity and duration of illness, personality, and other pre-treatment patient characteristics, (3) devising RCTs exploring the effectiveness of CRT for adolescents, using age appropriate instruments taking into account the heterogeneity in neuropsychological functioning in young individuals with AN (e.g. instruments assessing metacognitive abilities, self-awareness, motivation, ability and willingness to change, therapeutic alliance, quality of life and everyday functioning), (4) exploring of the applicability and effectiveness of transdiagnostic versions of CRT in larger samples, and finally, (5) the continuous investigation of family CRT as a conjunctive treatment for young girls with AN.

## Abbreviations

ADHD: Attention deficit hyperactivity disorder
AN: Anorexia nervosa
AN-EXRP: Exposure and response prevention for AN
BMI: Body mass index
BRIEF: Behavior rating inventory of executive function
CAT: Cognitive analytic therapy
CBT: Cognitive behavioral therapy
CREST: Cognitive remediation therapy and emotional skills training
CRT: Cognitive remediation therapy
DSM-5: The diagnostic and statistic manual of mental Disorders Fifth Edition
ED: Eating disorder
EF: Executive function
EDNOS: Eating disorder not otherwise specified
FBT: Family based therapy
IPT: Interpersonal therapy
NNT: Non-specific neurocognitive therapy
OCD: Obsessive compulsive disorder
RCT: Randomised controlled trial
TAU: Treatment as usual
TMT: Trail making test
